# Perceived Impact of Outdoor Swimming on Health: Web-Based Survey

**DOI:** 10.2196/25589

**Published:** 2022-01-04

**Authors:** Heather Massey, Paul Gorczynski, C Mark Harper, Lisa Sansom, Kieren McEwan, Alla Yankouskaya, Hannah Denton

**Affiliations:** 1 Extreme Environments Laboratory, School of Sport, Health and Exercise Science University of Portsmouth Portsmouth United Kingdom; 2 School of Sport, Health and Exercise Science University of Portsmouth Portsmouth United Kingdom; 3 Department of Anaesthesia Royal Sussex County Hospital Brighton United Kingdom; 4 Department of Anaesthesia Sørlandet Sykehus Kristiansand Norway; 5 Public Health Department, East Sussex County Council Lewes United Kingdom; 6 NHS Primary Care Hastings United Kingdom; 7 Department of Psychology Faculty of Science and Technology Bournemouth University Bournemouth United Kingdom; 8 West Recovery Team, Mill View Hospital Sussex Partnership NHS Foundation Trust Hove United Kingdom

**Keywords:** open water swimming, blue space, blue gym, mental health, physical health

## Abstract

**Background:**

Outdoor swimming in lakes, lidos (outdoor pools), rivers, and the sea has grown in popularity in many countries, including the United Kingdom. Many anecdotal accounts indicate improvements in medical conditions, which are considered a consequence of outdoor swimming.

**Objective:**

The aim of this study is to better understand outdoor swimmers’ perceptions of their health and the extent to which participation impacted their existing self-reported symptoms.

**Methods:**

A survey was conducted to investigate outdoor swimming behaviors and reports of any diagnosed medical conditions. Medical conditions were coded into categories, and descriptive statistics were generated regarding the outdoor swimmers’ behaviors and the effect that outdoor swimming had on their medical symptoms if any. The medical categories were clustered into five larger categories based on their prevalence in the current sample: mental health; musculoskeletal and injury; neurological; cardiovascular and blood disease; and *other*, which comprises inflammatory, immune, endocrine, and respiratory conditions.

**Results:**

In total, 722 outdoor swimmers responded, of whom 498 (68.9%) were female. The probability of outdoor swimming having *some positive impact* on health across all medical categories was 3.57 times higher compared with *no* impact (*B*=1.28, 95% CI 0.63-1.91; *P*<.001), 44.32 times higher for the mental health category (*B*=3.79, 95% CI 2.28-5.30; *P*<.001), 5.25 times higher for musculoskeletal and injury category (*B*=1.66, 95% CI 0.52-2.79; *P*=.004), and 4.02 times higher for the *other* category (*B*=1.39, 95% CI 0.27-2.51; *P*=.02). Overall, outdoor swimming was associated with perceived reductions in symptoms of poor mental health (*χ^2^*_2_=25.1; *P*<.001), musculoskeletal and injury (*χ*^2^_2_=8.2; *P*=.04), cardiovascular and blood (*χ*^2^_2_=14.7; *P*=.006), and *other* conditions (*χ*^2^_2_=18.2; *P*<.001).

**Conclusions:**

Physical activity in the form of outdoor swimming is perceived to have positive impacts on health and is associated with perceived symptom reductions in mental health, musculoskeletal and injury, and cardiovascular and blood conditions. This study cannot provide causal relationships or provide mechanistic insights. However, it does provide a starting point for more targeted prospective intervention research into individual conditions or categories of conditions to establish the impact in those who choose to start outdoor swimming.

## Introduction

### Background

Swimming outdoors is an increasingly popular recreational physical activity both in the United Kingdom and abroad [1,2], offering opportunities to be physically active in a range of facilities from natural water sources such as ponds, lakes, rivers, and the sea to man-made outdoor facilities, such as open-air pools or lidos. These locations differ from indoor pool swimming as they are based in natural environments, with lower water temperatures and fresh or salt water without chlorine treatment. These different attributes provide opportunities for those who prefer a more natural environment, those who cannot or prefer not to swim in chlorinated water, and those with limited access to indoor facilities.

Most research into exercise for health and well-being has been land-based. However, findings from a small number of swimming studies suggest that psychological effects are similar to exercise on land [3]. In addition, it has been suggested that bodies of difference can be enabled through immersion in water [4]. This can lead to a transformation of the *unhealthy* land body, for example, *large* and *middle-aged*, into a healthy sea body [5] and enable older people to challenge perceptions of burden and dependency [6].

The expansion of outdoor swimming has also been mirrored by the increased volume of research on the potential benefits of activity in blue spaces [7], thus, highlighting not only the need to remain physically active but also the potential of the natural environment to support improvements in health and well-being [8]. At this stage, there are many accounts suggesting that outdoor swimming can promote healthy aging and improve health [9,10]. The evidence remains at an anecdotal, case report or expert opinion level in accordance with evidence-based medicine criteria [11]. The accounts frequently discuss similar themes of transformation, connectedness, and reorientation, which have been well described by Denton and Aranda [12].

### Objective

The concept of cold water swimming or cold water spa treatments is not new; Hippocrates claimed that water therapy reduced lassitude (ie, lethargy) [13]. Considering the increasing volume of contemporary anecdotal evidence, we are no closer to establishing which medical conditions, if any, may be improved through regular outdoor swimming, how much improvement can be made, and by what mechanisms improvements occur. This research aims to provide a small step in that process by surveying outdoor swimmers to establish the medical conditions they have been diagnosed with and if they have perceived any change in their symptoms since starting to swim outdoors. Therefore, it is hypothesized that the type of medical condition can reliably predict the perceived health impact of outdoor swimming.

## Methods

### Survey Methods

The survey and manuscript were prepared in accordance with the Checklist for Reporting Results of Internet E-Surveys (CHERRIES) guidance [14]. A 14-item web-based cross-sectional survey was completed by 722 people who swim outdoors (duplicate and incomplete forms were removed) following ethical approval from the University of Portsmouth Science Faculty Research ethics committee (SFEC 2018-120), using the JISC web-based survey platform.

### Participants and Procedures

**Figure 1:**
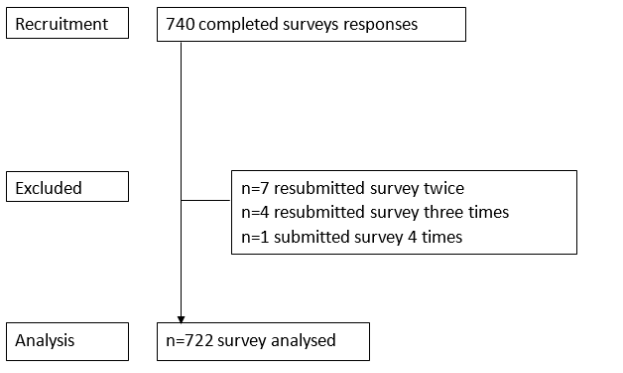
CONSORT (Consolidated Standards of Reporting Trials) diagram.

Participants were 722 outdoor swimmers (498/722, 68.9% female; 159/722, 22% male; and 65/722, 9% did not identify sex; outdoor swimmers are described here as participants who swim or immerse themselves in natural water environments, such as the sea, rivers, or lakes, or open-air pools). They gave their informed consent to allow the anonymous use of their data for the explicit purpose of establishing what medical conditions outdoor swimmers have and whether they gain any relief or symptom reduction from the activity. All participants freely volunteered to participate without incentive and were recruited using a snowball sampling approach using extended contact networks and social media [15]. Participants were told of the length of the survey, and the research lead’s name and contact details were displayed on the first and final pages. Figure 1 shows the pathway used in the study. The data were stored on password-protected servers at the University of Portsmouth.

### Instrument

The full survey was available on the web between November 1, 2018, and March 30, 2019, and advertised in Outdoor Swimmer magazine, which is a magazine for people who enjoy outdoor swimming. Once the survey had closed, duplicate responses were removed by using IP addresses and time stamps (30 surveys) and then data were deidentified from the IP address.

Participants voluntarily completed a web-based open survey hosted on a secure web-based survey platform with automatic back end data capture. Participants were asked to answer open-ended questions related to their participation in outdoor swimming. They identified where they swam, whether in outdoor facilities (lido or open-air pool) or in a natural setting (eg, the sea, lakes, and rivers), when they swam outdoors, their regularity of outdoor swimming, and their initial motives for taking up open water swimming. They were then asked to identify the physical and mental health conditions they had been diagnosed with, the impact and change in symptoms that were experienced while engaged in outdoor swimming, whether their symptoms had changed since outdoor swimming, and how long their symptoms changed for. There was no randomization of the survey items. Participants were able to press a back button to review their answers or resubmit their answers. A nonresponse option was also included in each question.

### Pilot Testing

A pilot survey (n=10) was conducted between September 15, 2018, and October 20, 2018, to evaluate the instrument length, language, and logic. Modifications were made to the language, and additional answer options were added to closed questions to improve participant understanding and response rate.

### Data and Statistical Analyses

Once duplicate responses had been removed, the data set was deidentified. Medical conditions were retrospectively coded using the health categories from the UK Clinical Research Collaboration [16]. Coding was initially undertaken by the corresponding author and cross-referenced with 2 practicing medical doctors (MH and LS). All conditions were coded, leading to some participants having multiple codes because of comorbidities. In a number of cases, no conditions were reported, or the conditions could not be categorized. In these cases, 2 additional categories were used: *no condition* and *not categorized*. The latter occurred primarily because of a vague description of symptoms (out of the 1084 conditions categorized, it occurred in a small number; n=30, 2.77%).

The 21 health condition categories were clustered into five main areas: (1) mental health; (2) injury, accidents, and musculoskeletal injuries; (3) neurological; (4) cardiovascular and blood; and (5) other. These 5 groups were chosen as they encompassed most of the responses. Many who were classified in the injury and accidents group were also classified into the musculoskeletal injuries group. Similarly, cardiovascular conditions were combined with blood disorders, as participants were frequently categorized in both separately. The *other* category contains the inflammatory and immune, metabolic and endocrine, and respiratory conditions clustered together because of the small number in the individual categories. Although there may be associations with this category as a whole, this may not translate into associations or impacts in all conditions within this group. The medical conditions contained within the *other* category are diverse, and for many of the conditions in this group (cancer, congenital disorders, and renal conditions), there are no anecdotal reports that the authors are aware of that indicate improvements in symptoms as a consequence of outdoor swimming.

**Figure 2:**
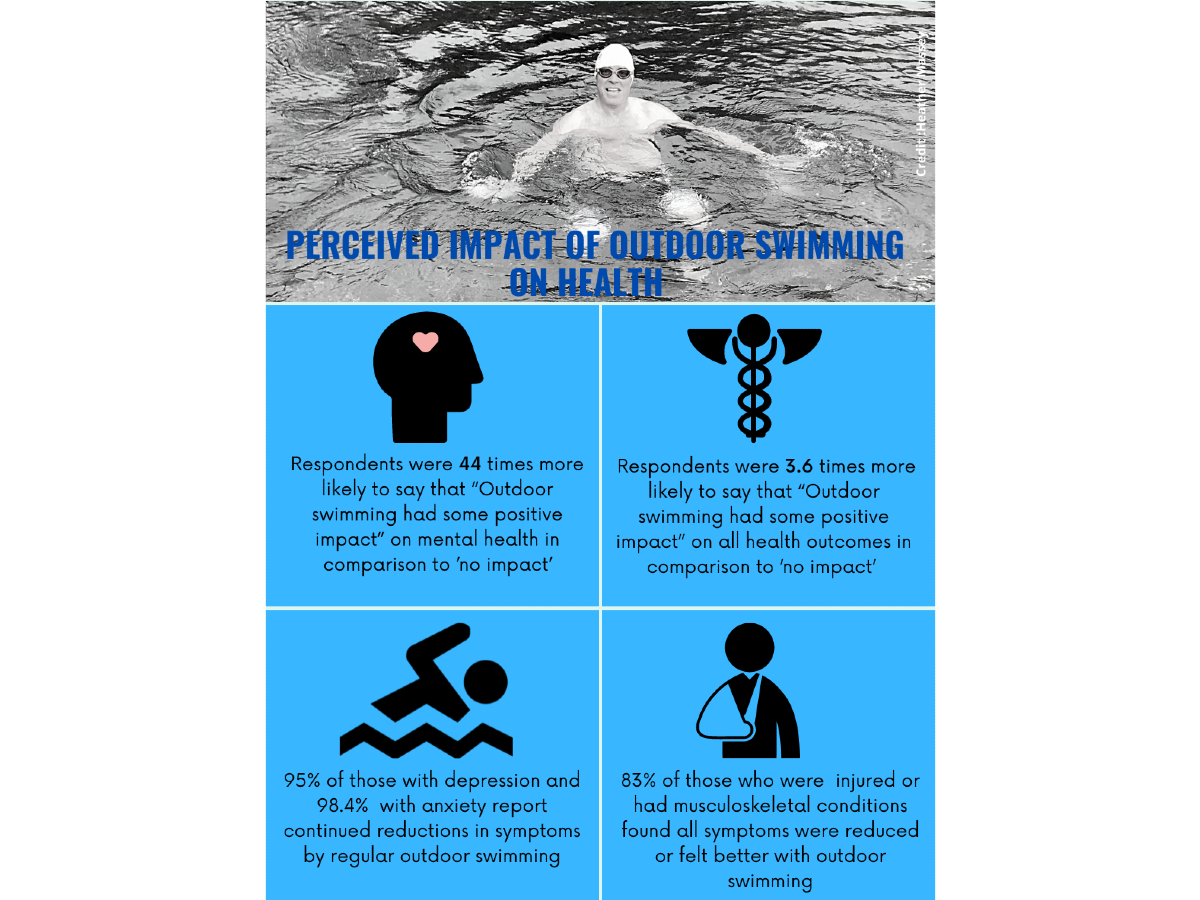
Study infographic. Image credit: Heather Massey.

The perceived impact of outdoor swimming was measured on a 4-point scale (1=a lot of impact, 2=some impact, 3=little impact, and 4=no impact). Logistic regression analysis was used to test whether participants’ medical categories could predict the perceived impact of outdoor swimming. The medical categories were clustered into 5 larger categories based on their prevalence in the current sample: mental health, musculoskeletal and injury, neurological, cardiovascular and blood disease, and *other*. These categories were entered into the analysis as predictors, and regression coefficients were estimated based on a bootstrapping procedure with 5000 successful replicates [17]. Logistic regression analyses were performed using the R-based statistical software JASP (JASP version 0.13.1). A detailed analysis and model fit check can be found in Figure 2.

Other statistical analyses were conducted using SPSS (version 25; IBM Corp). The frequency of responses per medical category was tabulated, and further chi-square analyses were performed in those medical categories that had at least 5% of the participants’ self-reports. Chi-square tests of association analyzed the gender; regularity; impact of outdoor swimming on their conditions (both reduced and increased severity of symptoms); the change in symptoms, if any, resulting from outdoor swimming; and finally, whether the regularity of outdoor swimming was associated with changes in symptoms. For all tests of association, statistical significance was defined as *P*<.05. The strength of association was considered using Cramer *V*, based on the following thresholds: small=0.1, moderate=0.3, and large=0.5 [18].

## Results

### Participant Characteristics

A total of 722 separate participants entered the survey analysis (Figure 1). Most of the participants were female (498/722, 68.9%), with further participants being male (159/722, 22%) or not stating sex (65/722, 9%). All participants swam outdoors in open water (lakes, rivers, the sea, lochs, quarries, lidos, and reservoirs). They reported swimming all year round (487/722, 67.5%) or seasonally in the summer and autumn (151/722, 20.9%) or only in the winter (1/722, 0.1%). A further group had recently taken up outdoor swimming and were not sure how long they would continue swimming once the water started to cool (58/722, 8%). The main reason for starting outdoor swimming included training for an event or challenge (138/722, 19.1%), liking to feel connected with nature (106/722, 13.7%), improved well-being (98/722, 13.6%), started as a child (70/722, 9.7%) and by a friend’s invite (64/722, 8.9%), a change from pool swimming (36/722, 4.9%), and not sure (37/722, 5.1%).

### Medical Category Prevalence

The number of respondents also reported multiple medical conditions, and the frequency of reports of single and comorbid conditions are reported in Table 1. A frequency table of the medical categories reported by the participants is shown in Table 2. Most medical conditions reported were in the mental health category (399/722, 55.3%), followed by musculoskeletal (153/722, 21.2%), no medical condition (76/722, 10.5%), neurological conditions (66/722, 9.1%), cardiovascular (61/722, 8.4%), respiratory (52/722, 7.2%), metabolic and endocrine (45/722, 6.2%), and finally, inflammatory or immune conditions (38/722, 5.3%). These medical categories are the main focus of further analysis.

Sex was significantly associated with the categories *no medical condition* (*χ*^2^_2_=11.2; *P*=.004) and neurological conditions (*χ*^2^_2_=7.3; *P*=.03). More males were associated with no conditions and more females with neurological conditions. No other medical categories had any association with sex.

The duration of symptoms before outdoor swimming was associated with mental health (*χ*^2^_6_=84.6; *P*<.001), musculoskeletal and endocrine (*χ*^2^_6_=19.4; *P*=.004), and respiratory categories (*χ*^2^_6_=19.5; *P*=.003). All were associated with long-term symptoms exceeding 10 years.

**Table 1:** Frequencies, reporting a single medical category, multiple medical conditions, or no medical condition (N=722).

Conditions	Values, n (%)
No condition	76 (10.5)
Single condition	401 (55.5)
Comorbid	245 (33.9)

**Table 2:** Frequency of medical conditions reported, percentage of responses, and percentage of participants reporting each medical category (from the UK Clinical Research Collaboration [16]).

Conditions	Responses (N=1084), n (%)	Participants (N=722^a^), n (%)
Not able to categorize	30 (2.8)	30 (4.2)
None	76 (7)	76 (10.5)
Blood	7 (0.6)	7 (1)
Cancer	13 (1.2)	13 (1.8)
Cardiovascular	61 (5.6)	61 (8.4)
Congenital disorder	7 (0.6)	7 (1)
Ear	2 (0.2)	2 (0.3)
Eye	4 (0.4)	4 (0.6)
Infection	14 (1.3)	14 (1.9)
Inflammatory and immune system	38 (3.5)	38 (5.3)
Injury and accidents	18 (1.7)	18 (2.5)
Mental health	399 (36.9)	399 (55.3)
Metabolic and endocrine	45 (4.2)	45 (6.2)
Musculoskeletal	153 (14.2)	153 (21.2)
Neurological	66 (6.1)	66 (9.1)
Oral gastrointestinal	17 (1.6)	17 (2.4)
Renal and urogenital	6 (0.6)	6 (0.8)
Reproductive health and childbirth	19 (1.8)	19 (2.6)
Respiratory	52 (4.8)	52 (7.2)
Skin	26 (2.4)	26 (3.6)
Stroke	0 (0)	0 (0)
Generic health relevance	0 (0)	0 (0)
Other	31 (2.9)	31 (4.3)

^a^The participant percentage will exceed 100% because of a number of participants reporting comorbidities.

### Impact of Outdoor Swimming

**Figure 3:**
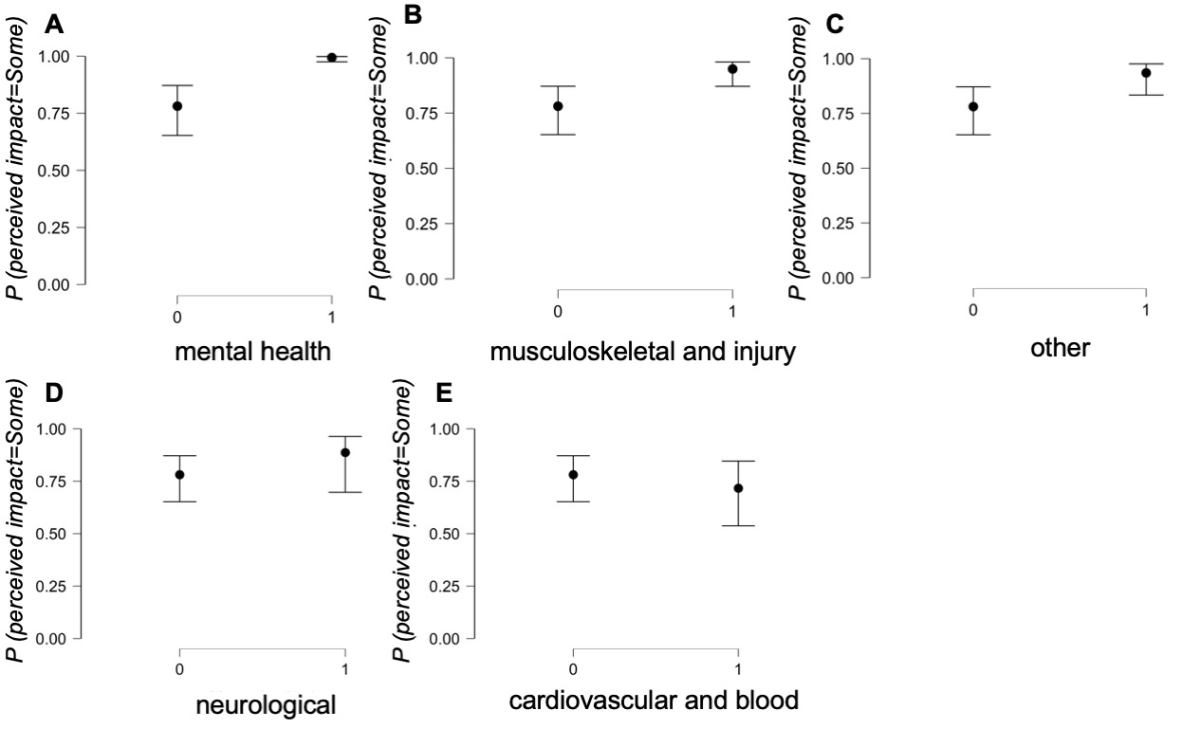
Condition estimate plots. The probability of having impact of outdoor swimming on health (y-axis) plotted against responses no impact (denoted as 0) and some impact (denoted as 1) for 5 categories of medical conditions. Black dots represent the probability of impacts. The bars represent 95% CIs.

Approximately 89.6% (647/722) of participants reported at least one medical condition; their responses to the question “has open water swimming had any impact on your medical symptoms” established that 95.5% (618/647) found *some positive impact,* and 4.5% (29/647) found *no impact*. The logistic regression model was statistically significant (*χ*^2^_637_=60.8; *P*<.001; McFadden *R^2^*=0.26). The model’s intercept indicated that the probability of having *some positive impact* of outdoor swimming on health across all medical categories was 3.57 times higher than that of *no* impact (*B*=1.27, 95% CI 0.63-1.91; Wald statistics=15.13, degree of freedom=1; *P*<.001). The probability of having *some positive impact* of outdoor swimming on health was 44.32 times higher for the mental health category (*B*=3.79, 95% CI 2.28-5.30; Wald statistics=24.36, degree of freedom=1; *P*<.001), 5.25 times higher for the musculoskeletal and injury category (*B*=1.66, 95% CI 0.52-2.79; Wald statistics=8.26, degree of freedom=1; *P*=.004), and 4.02 times higher for the *other* category (*B*=1.39, 95% CI 0.27-2.51; Wald statistics=5.90, degree of freedom=1; *P*=.02; Figure 2; Table 3). For neurological (*B*=0.78, 95% CI −0.50 to 2.08; Wald statistics=1.42, degree of freedom=1; *P*=.23) and cardiovascular and blood (*B*=−0.34, 95% CI −1.27 to 0.58; Wald statistics=0.54, degree of freedom=1; *P*=.46) categories, the estimates were not statistically significant (Figures 2 and 3).

**Table 3:** Frequency of medical conditions reported into collapsed medical categories, percentage of responses, and percentage of participants reporting each medical category (N=917).

Conditions	Responses, n (%)	Participants, n (%)
Mental health	399 (43.5)	399 (62.7)
Injury and accidents and musculoskeletal	165 (18)	165 (25.9)
Neurological	66 (7.2)	66 (10.4)
Cardiovascular and blood	67 (7.3)	67 (10.5)
Other	200 (24)	200 (34.6)

### Characterizing Impact

The reasons given for starting outdoor swimming were numerous; however, associations were found for the mental health and musculoskeletal and injury categories. The respondents’ reporting of mental health conditions indicated an association with starting outdoor swimming to train for an event or challenge (*χ*^2^_10_=36.1; *P*<.001). In contrast, those with musculoskeletal conditions or injuries initially participated in outdoor swimming as injuries prevented them from taking part in other forms of activity (*χ*^2^_10_=31.9; *P*<.001).

On further questioning, the perceived impact was a reduction in symptoms because of outdoor swimming in the following categories: mental health (*χ*^2^_3_=25.1; *P*<.001), musculoskeletal and injury (*χ*^2^_3_=8.2; *P*=.04), cardiovascular and blood (*χ*^2^_3_=14.7; *P*=.006), and *other* categories (*χ*^2^_3_=18.2; *P*<.001; Table 4). Furthermore, trends for a reduction in symptoms after open water swimming were reported in the mental health (*χ*^2^_1_=2.9; *P*=.08) and *other* categories (*χ*^2^_1_=2.7; *P*=.08); these symptom reductions in the mental health category were short-lived (*χ*^2^_4_=35.4; *P*<.001), lasting from several hours to 2 days after swimming outdoors. No other associations were found between the duration of reduced symptoms and the medical categories.

Considering the *dose of swimming or cold water*, no associations between swimming frequency and the impact or a change in symptoms were found in any medical category. The same is true of the choice of swimming attire (wetsuit or swimming costume). However, more respondents with cardiovascular and blood conditions wore wetsuits (*χ*^2^_2_=7.6; *P*=.21). In contrast, there was a trend for association in the *other* category, indicating that greater numbers of respondents found that their symptoms were affected if they swam all year round (*χ*^2^_3_=8.4; *P*=.10). In addition, for many in the musculoskeletal and injury (*χ*^2^_3_=9.8; *P*=.02), mental health (*χ*^2^_3_=10.0; *P*=.02), and *other* categories (*χ*^2^_3_=13.3; *P*=.04), the duration of swimming was primarily governed by the water temperature.

**Table 4:** Characteristics of the impact of outdoor swimming on the 5 combined medical categories. Percentages represent responses within each medical category (N=722).

Characteristics		Medical categories				
		Mental health (n=395)	Injury, accident, and musculoskeletal (n=161)	Neurological (n=63)	Cardiovascular and blood (n=62)	Other (n=207)
**Symptom changes because of swimming (n=622)**						
	All symptoms reduced or feel better, n (%)	360 (91.1)	134 (83.2)	50 (79.4)	44 (71)	167 (80.7)
	No change, n (%)	27 (6.8)	14 (8.7)	10 (15.9)	12 (19.4)	22 (10.6)
	All symptoms increased or feel worse, n (%)	0 (0)	3 (1.9)	0 (0)	1 (1.6)	3 (1.4)
	Some symptoms reduced some increased, n (%)	8 (2)	10 (6.2)	3 (4.8)	5 (8.1)	15 (7.2)
	Chi-square (*df*)	25.1 (3)	8.2 (3)	4.8 (3)	14.7 (3)	18.2 (3)
	*P* value	<.001^a^	.04^a^	.16	.006^a^	<.001^a^
	Cramer *V*	0.201	0.115	0.088	0.154	0.171
**Continued reduction in symptoms by open water swimming (n=594)**						
	Yes, n (%)	362 (96.5)	151 (95.6)	58 (65.1)	55 (98.2)	187 (93.5)
	No, n (%)	13 (3.5)	7 (4.4)	3 (4.9)	1 (1.8)	13 (6.5)
	Chi-square (*df*)	2.7 (1)	0.0 (1)	0.0 (1)	1.1 (1)	2.7 (1)
	*P* value	.08^b^	.57	.54	.26	.08^b^
	Cramer *V*	0.068	0.003	0.006	0.043	0.067
**If you have continued symptom reduction how long does this last for? (n=576)**						
	Several hours, n (%)	46 (12.3)	25 (16.3)	11 (18.3)	4 (7.7)	21 (11.4)
	1 to 2 days, n (%)	187 (50)	61 (39.9)	18 (30)	18 (34.6)	73 (39.7)
	1 week, n (%)	31 (8.3)	8 (5.2)	3 (5)	5 (9.6)	11 (6)
	>1 week, n (%)	49 (13.1)	38 (24.8)	13 (21.7)	14 (26.9)	41 (22.3)
	Cannot say, n (%)	61 (16.3)	21 (13.7)	15 (25)	11 (21.2)	38 (20.7)
	Chi-square (*df*)	35.4 (4)	8.4 (4)	6.9 (4)	5.7 (4)	5.0 (4)
	*P* value	<.001^a^	.08^b^	.14	.22	.29
	Cramer *V*	0.248	0.121	0.109	0.1	0.093
**Regularity of swimming (n=634)**						
	Daily, n (%)	39 (9.8)	16 (9.7)	10 (16.1)	6 (9.1)	23 (10.6)
	At least once a week, n (%)	317 (80.6)	133 (81.2)	43 (69.4)	55 (83.3)	171 (79.2)
	Less than once a week or infrequently, n (%)	37 (9.4)	15 (9.2)	9 (14.5)	5 (7)	22 (10.2)
	Chi-square (*df*)	0.2 (2)	0.0 (2)	5.9 (2)	0.3 (2)	0.7 (2)
	*P* value	.88	.99	.05^a^	.89	.71
	Cramer *V*	0.02	0.007	0.097	0.021	0.034

^a^Statistically significant at *P*≤.05.

^b^Trend to statistical significance (*P*≤.10).

### Prevalence of Mental Health Conditions and Impact of Outdoor Swimming

Respondents reporting mental health conditions were the largest cohort in this survey. Table 5 contains a breakdown of the main mental health conditions reported by the participants. Of interest, 30.8% (123/399) participants reported a diagnosis of depression and anxiety, with a further 20.1% (80/399) diagnosed with anxiety alone and 41.1% (164/399) with depression alone. Furthermore, depression was associated with a longer period since diagnosis (*χ*^2^_4_=11.7; *P*=.03). Despite a large numerical response in all conditions within the mental health category, no significant associations of the impact of outdoor swimming on symptoms of these conditions were found, except that a reduction in symptoms was associated with anxiety (*χ*^2^_3_=7.0; *P*=.03) and a trend for association with depression (*χ*^2^_3_=5.4; *P*=.09). In addition, reports of a continued reduction in symptoms were associated with diagnoses of anxiety or depression (anxiety *χ*^2^_1_=4.2, *P*=.04 and depression *χ*^2^_1_=5.7, *P*=.03). Therefore, 95% (251/264) of respondents with depression and 98.4% (188/191) of respondents with anxiety reported continued reductions in symptoms through regular outdoor swimming.

**Table 5:** Cross tabulations between mental health conditions and outdoor swimming survey item responses. Percentages have been calculated across all mental health conditions and responses.

Characteristics			Medical condition within the mental health category								
			Depression (n=287)		Anxiety (n=203)		Posttraumatic stress disorder (n=25)		Bipolar (n=11)		Other (n=40)
**Gender (n=399)**											
	Female, n (%)	212 (53.2)		149 (37.4)		14 (3.5)		9 (2.3)		28 (7)	
	Male, n (%)	53 (13.3)		34 (8.5)		9 (2.3)		2 (0.5)		9 (2.3)	
	None given, n (%)	22 (5.5)		20 (5)		2 (0.5)		0 (0)		3 (0.8)	
	Chi-square (*df*)	0.8 (2)		3.7 (2)		4.7 (2)		1.0 (2)		0.3 (2)	
	*P* value	.68		.16		.09^b^		.70		.92	
	Cramer *V*	0.045		0.097		0.109		0.051		0.027	
**Symptom duration (n=399)**											
	<1 year, n (%)	3 (0.8)		5 (1.3)		0 (0)		0 (0)		2 (0.5)	
	1 to 4.9 years, n (%)	44 (11)		32 (8)		3 (0.8)		0 (0)		10 (2.5)	
	5 to 9.9 years, n (%)	18 (4.5)		21 (5.3)		1 (0.3)		1 (0.3)		1 (0.3)	
	≥10 years, n (%)	197 (49.4)		135 (33.5)		20 (5)		9 (2.3)		24 (6)	
	Not sure, n (%)	24 (6)		10 (2.5)		1 (0.3)		1 (0.3)		3 (0.8)	
	Chi-square (*df*)	11.7 (4)		9.2 (4)		2.8 (4)		2.5 (4)		6.7 (4)	
	*P* value	.03^a^		.08^b^		.63		.66		.24	
	Cramer *V*	0.171		0.152		0.084		0.08		0.129	
**Impact of swimming on condition (n=397)**											
	Yes, n (%)	284 (71.4)		201 (50.8)		25 (6.3)		11 (2.8)		39 (9.8)	
	No, n (%)	2 (0.5)		1 (0.3)		0 (0)		0 (0)		0 (0)	
	Chi-square (*df*)	0.8 (1)		<0.1 (1)		0.1 (1)		<0.1 (1)		0.2 (1)	
	*P* value	.59		.74		.88		.95		.81	
	Cramer *V*	0.044		0.001		0.018		0.012		0.024	
**Symptom changes because of swimming (n=395)**											
	All symptoms reduced or feel better, n (%)	255 (64.6)		188 (47.7)		24 (6.1)		11 (2.8)		36 (9.1)	
	No change, n (%)	24 (6.1)		7 (1.8)		0 (0)		0 (0)		3 (0.8)	
	All symptoms increased or feel worse, n (%)	0 (0)		0 (0)		0 (0)		0 (0)		0 (0)	
	Some symptoms reduced some increased, n (%)	5 (1.3)		4 (1)		1 (0.3)		0 (0)		0 (0)	
	Chi-square (*df*)	5.4 (3)		7.0 (3)		2.4 (3)		1.1 (3)		0.1 (3)	
	*P* value	.08^b^		.03^a,^		.31		.70		.75	
	Cramer *V*	0.106		0.134		0.078		0.053		0.049	
**Continued reduction in symptoms by regular open water swimming (n=391)**											
	Yes, n (%)	251 (69.3)		188 (50.3)		24 (6.4)		9 (2.4)		38 (10.2)	
	No, n (%)	13 (3.5)		3 (0.8)		0 (0)		0 (0)		0 (0)	
	Chi-square (*df*)	5.6 (1)		4.2 (1)		0.9 (1)		0.3 (1)		1.5 (1)	
	*P* value	.03^a^		.04^a^		.42		.72		.24	
	Cramer *V*	0.123		0.106		0.05		0.03		0.064	
**If you continued swimming, how long were symptoms reduced? (n=373)**											
	Hours, n (%)	127 (34)		76 (20.4)		9 (2.4)		1 (1)		11 (2.9)	
	1 to 2 days, n (%)	73 (19.5)		60 (16.1)		9 (2.4)		2 (0.5)		12 (3.2)	
	1 week, n (%)	18 (4.8)		18 (4.8)		2 (0.5)		2 (0.5)		4 (1.1)	
	>1 week, n (%)	10 (2.7)		9 (2.4)		0 (0)		0 (0)		1 (0.3)	
	Cannot say, n (%)	42 (11.2)		27 (7.2)		5 (1.3)		3 (0.8)		10 (2.7)	
	Chi-square (*df*)	5.7 (4)		9.6 (4)		2.2 (4)		2.9 (4)		5.5 (4)	
	*P* value	.22		.04^a,^		.70		.51		.23	
	Cramer *V*	0.123		0.161		0.078		0.089		0.121	

^a^Statistically significant at *P*≤.05.

^b^Trend to statistical significance, *P*≤.10.

## Discussion

### Principal Findings

The main findings suggest that physical activity in the form of outdoor swimming affects health and is associated with perceived improvements in some medical categories (mental health, musculoskeletal and injury, and *other*) but not all. The level of evidence at present remains anecdotal [11]. However, by performing the survey, it is clear which categories of medical conditions may have perceived benefits from outdoor swimming. Consequently, these data can be used to focus future research efforts in the conditions where anecdotal support was apparent to establish causality and, if so, potential mechanisms for symptom reduction.

Just over half (399/722, 55.3%) of the survey respondents reported a diagnosed mental health condition. This proportion is higher in comparison with the general population (before the COVID-19 lockdown, 9.7% and after the COVID-19 lockdown, 19.7% [19]), and it is unknown whether this is representative of the population of outdoor swimmers. The use of a single category to describe mental health conditions is limiting. Further subcategorization was possible in this study, with associations between a perceived reduction in symptoms in respondents’ reporting of anxiety and depression. In contrast, there is insufficient evidence to suggest a reduction in symptoms of other mental health conditions, which may be because of the smaller number of respondents with these conditions in the present survey. Case report evidence exists of outdoor swimming as a nonmedicalized activity, supporting recovery from depression and anxiety [9]. It is also unclear whether outdoor swimming would result in symptom reduction in all forms of depression or the potential mechanisms at play [20]. In addition, it may also be that symptoms are not changed by the act of outdoor swimming, but the participants’ perceptions of their symptoms or feelings of well-being are temporarily changed. However, it is important to separate well-being from mental health rather than them occupying the same continuum [21]. Therefore, it may be that outdoor swimming does not reduce their symptoms, but they do have a sense of greater well-being. Consequently, this study and qualitative aspects of the survey data that are yet to be published (McEwan, personal communication) provide a focus to build research designs that can establish causality and potentially the mechanisms involved.

The benefits of physical activity are well-documented and include a reduced risk of developing both physical and mental health problems and can support the treatment of pre-existing health conditions [22,23]. It is also acknowledged that exercise close to coastal areas or *blue spaces* may affect well-being [24] and mood [25]. Similar to terrestrial activity, swimming appears to positively affect markers of ill health [26], and swimming also offers the opportunity for reduced load bearing on muscles and joints [27]. This may support an explanation for the perception of reduced symptoms or pain in those with musculoskeletal conditions such as arthritis and back pain who do not feel able to be active on land [28]. Greater movement and pain reduction were also reported during immersion in cold water [29]. Therefore, for participants with musculoskeletal problems, outdoor swimming may allow greater movement, activity level, and respite from pain than is possible on land; however, further research is needed to confirm this hypothesis.

Outdoor swimming, for many of the survey respondents has become a lifestyle choice, opting to be frequently physically active (at least once a week) in outdoor water environments. Overall, there was no association between the frequency of swims, swim attire, or swim duration and the impact or reports of symptom reduction. However, there was a trend for association between having an impact and swimming all year round in the *other* category. In addition, the duration of swimming was associated with the water temperature, weather, and sea state. Longer duration swims take place in warmer, calmer water, and shorter swims take place in cooler, rougher water. Therefore, the dose of outdoor swimming cannot be easily prescribed and will depend on the individuals’ physiological responses to immersion in cold water, the location, sea state or moving water, and weather conditions. Cold water swimming is not a risk-free activity; individuals with underlying cardiac and cardiovascular conditions may be at elevated risk of adverse cardiac events upon initial immersion in cold water, and those who are unable to keep their airway clear of the water are at risk of drowning because of the cold shock response [30]. Therefore, consultation with a general practitioner is recommended for those wanting to try outdoor swimming and have an underlying medical condition. It is also recommended that those new to outdoor swimming join a group or swim with a trained open water swimming coach with good local knowledge of the environment and are able to convey the knowledge and skills required to swim safely outdoors.

In this survey, respondents provided information on their motives for starting to swim outdoors. These motives ranged from training for an event to looking for a change from a swimming pool, with some individuals not being sure why they started. Some people clearly stated that it was to improve the elements of their mental health. As seen in other research studies [12], there are several reasons why people begin to and maintain swimming outdoors. Although we may better understand some of these motives, we still do not know whether swimming outdoors has medical utility. Before any recommendations can be made as to the health effects of swimming outdoors, the evidence must clearly demonstrate both association and causation with swimming outdoors and improved health [31]. This research forms part of the development process and our continuing research efforts to better understand and demonstrate evidence of the health impact of outdoor swimming.

### Limitations

It is acknowledged that the survey was conducted on people currently swimming in open water, and therefore, those who are likely to have a very positive viewpoint about the activity. Although the views expressed in this paper and in the sister paper have been commonly heard by the authors, it is not clear if this is representative of all outdoor swimmers or those who do not continue to swim outdoors. However, the research was conducted as an internet-based open survey; therefore, no coercion took place for swimmers to participate or give particular answers. In addition, duplicate submissions were removed to prevent the overrepresentation of one person’s viewpoint. Furthermore, there may be participants who feel compelled to support outdoor swimming for health improvement. However, most swimmers did not start outdoor swimming to find a means of improving health, but many self-reported health improvements as a consequence of their swimming.

The survey grouped medical conditions into categories, which has limitations in terms of specificity to individual conditions. However, the vast range of potential disease conditions made this the most practical step to take. There were generally a small number of conditions that were more common; for example, in the mental health category, diagnoses of depression and anxiety were common and have been interrogated more deeply than other categories. Similarly, migraine was the most frequent condition in the neurological category. In medical categories with small numbers, this should not rule out any impact that the activity may have on their symptoms, and the number of participants with these conditions was too small to provide a statistical association. The survey as an initial investigation is adequate to gather together anecdotal self-reports; however, further, more in-depth investigation is required. Potential avenues for progressing the research include corroborating patient-reported outcomes with independent medical judgment and inclusion of condition-specific surveys to assess the patient-reported outcomes in a more sensitive, specific, and systematic manner. However, this approach now seems more feasible and can be better targeted, given the information in this paper.

The type of medical condition may limit some of the given responses; for instance, migraine attacks are sporadic and not easy for people to determine how severe or long an attack would last or even if swimming in cold water caused a reduction in symptoms. However, the participants may still have future migraines; therefore, outdoor swimming has not *cured* the condition. Therefore, although they may report some acute symptom reduction, it is unclear how frequently they might or will have these attacks, and without detailed logs of attacks and severity scoring, it can be challenging to establish whether the intervention, in this case, outdoor swimming, is affecting their symptoms.

### Opportunities for Further Study

It is well-established that people’s perceptions of their illness are crucial to health care providers to evaluate and support the patient’s empowerment and self-care ability. An individual’s perception of factors related to their illness or symptoms influences their coping behavior [32], trust in themselves, beliefs that they can manage their illness and prevent it from becoming worse [33]. Therefore, an individual’s experience in noticing and interpreting their experience of bodily changes during self-care activities such as engaging in outdoor swimming is important information for health care providers about the efficiency of such activities in promoting self-care. Furthermore, a person’s perception of improvement in health has a positive effect [34]. This relationship is conceptualized in the Common Sense Model of Illness Perception, which proposes that the positive beliefs that a person holds regarding their illness directly lead to better mental well-being and the development of active coping strategies [35]. However, validation of perceived improvements in health would need clinical interventions that would provide invaluable information about alternative methods for treating many physical and mental health conditions. Therefore, further research is needed to establish whether swimmers’ perceptions of improved health translate into outdoor swimming being effective in reducing symptoms of poor mental and physical health. Potential research should look to establish the clinical and cost-effectiveness of outdoor swimming as a treatment intervention. In addition, it would also need to fall in line with current policies such as the National Health Service Long-Term Plan [36], Mental Health Implementation Plan [37], the Community Mental Health Framework for Adults and Older Adults [38], and the Garside report [39]. These policies recommend the need for personalized care and patient choice and provide a framework for delivery. In particular, the Garside report [39] describes the limited evidence base for nature-based activities. So far, the main focus has been on land-based activities, with few water-based interventions. Therefore, further studies to establish the impact on mental and physical health conditions could use clinical trial methodologies. For instance, these may include randomized control trials with mixed method approaches to establish whether the outdoor swimming intervention is acceptable and has a therapeutic effect. If a therapeutic effect is found, for whom does it have an effect (in terms of a medical condition, patient age, and socioeconomic status), and how does that effect occur (through psychological, sociological, or physiological mechanisms, or more likely a combination of all 3)? Further avenues for research into the perception of impact on health conditions may be considered, such as *why participants perceive improvements*. Such research may include the use of innovative qualitative study methods; one such method was explored with great effect by Denton and Aranda [12].

### Conclusions

In conclusion, physical activity in the form of outdoor swimming was perceived to have a positive impact on health and is associated with perceived improvements in some medical conditions, namely mental health, musculoskeletal, and cardiovascular conditions. For many, not just those reporting a reduction in symptoms of a medical condition, outdoor swimming has become a lifestyle choice to be physically active in cold water. Although this study cannot provide causal relationships, unpick the reason for symptom reduction, or provide mechanistic insight, it does provide a starting point for more targeted research into individual conditions or categories of conditions in those who choose or would like to start outdoor swimming.

## Abbreviations

CHERRIES: Checklist for Reporting Results of Internet E-Surveys
